# *De Novo* Assembly of the Japanese Flounder (*Paralichthys olivaceus*) Spleen Transcriptome to Identify Putative Genes Involved in Immunity

**DOI:** 10.1371/journal.pone.0117642

**Published:** 2015-02-27

**Authors:** Lin Huang, Guiyang Li, Zhaolan Mo, Peng Xiao, Jie Li, Jie Huang

**Affiliations:** 1 Key Laboratory of Experimental Marine Biology, Institute of Oceanology, Chinese Academy of Sciences, Qingdao, China; 2 Key Laboratory of Sustainable Development of Marine Fisheries, The Ministry of Agriculture, Yellow Sea Fisheries Research Institute, Chinese Academy of Fishery Sciences, Qingdao, China; 3 National Laboratory for Marine Science and Technology, Qingdao, China; 4 University of Chinese Academy of Sciences, Beijing, China; 5 College of Life Science, Qingdao University, Qingdao, China; The National Orchid Conservation Center of China; The Orchid Conservation & Research Center of Shenzhen, CHINA

## Abstract

**Background:**

Japanese flounder (*Paralichthys olivaceus*) is an economically important marine fish in Asia and has suffered from disease outbreaks caused by various pathogens, which requires more information for immune relevant genes on genome background. However, genomic and transcriptomic data for Japanese flounder remain scarce, which limits studies on the immune system of this species. In this study, we characterized the Japanese flounder spleen transcriptome using an Illumina paired-end sequencing platform to identify putative genes involved in immunity.

**Methodology/Principal Findings:**

A cDNA library from the spleen of *P. olivaceus* was constructed and randomly sequenced using an Illumina technique. The removal of low quality reads generated 12,196,968 trimmed reads, which assembled into 96,627 unigenes. A total of 21,391 unigenes (22.14%) were annotated in the NCBI Nr database, and only 1.1% of the BLASTx top-hits matched *P. olivaceus* protein sequences. Approximately 12,503 (58.45%) unigenes were categorized into three Gene Ontology groups, 19,547 (91.38%) were classified into 26 Cluster of Orthologous Groups, and 10,649 (49.78%) were assigned to six Kyoto Encyclopedia of Genes and Genomes pathways. Furthermore, 40,928 putative simple sequence repeats and 47, 362 putative single nucleotide polymorphisms were identified. Importantly, we identified 1,563 putative immune-associated unigenes that mapped to 15 immune signaling pathways.

**Conclusions/Significance:**

The *P. olivaceus* transciptome data provides a rich source to discover and identify new genes, and the immune-relevant sequences identified here will facilitate our understanding of the mechanisms involved in the immune response. Furthermore, the plentiful potential SSRs and SNPs found in this study are important resources with respect to future development of a linkage map or marker assisted breeding programs for the flounder.

## Introduction

Japanese flounder (*Paralichthys olivaceus*) is an economically important marine fish that has been widely cultured in Asian countries, especially China, Korea, and Japan [1]. In fact, the gross production of this species amounts to 30,000 tons annually in China. However, increasing industrial farming has rendered Japanese flounder susceptible to various pathogens, including viruses [2], bacteria [3], and parasites [4], which have resulted in severe infectious diseases and heavy economic losses. To reduce the impact of disease, knowledge about the immune system and defense mechanisms against pathogens in this fish species is essential for the establishment of effective measures in disease control. For examples, the identification of immune-relevant genes and pathways in flounder could help in the development of potential markers for disease resistance, thereby allowing the establishment a successful genetic breeding program. Moreover, immune molecules, such as antimicrobial peptides and cytokines, present novel anti-microbial drugs and immunomodulatory adjuvants. Previous studies have been limited to discover and identify new immune-relevant genes from cDNA libraries [5, 6], although several immune-relevant genes have been identified and characterized, such as genes encoding T cell receptors (TCRs) [7], the cytosolic sensor gene (MDA5) [8], interferon regulatory factor 9 (IRF9) [9], and ATP-gated P2X7 receptor [10]. However, only one immune gene was developed for marker-assisted selective breeding in the flounder [11]. Therefore, a fast and cost-effective approach to identify amounts of immune genes for flounder is required.

Next-generation sequencing technology can generate large amounts of sequence data for a given organism at affordable costs, and high throughput RNA sequencing (RNA-Seq), including Solexa/Illumina, Roche/454 and ABI/SOLID, have generated transcriptomic and genomic resources. Using this sequencing technique, a large number of immune-related genes in several fish species were identified, including turbot (*Scophthalmus maximus*) [12], Japanese seabass (*Lateolabrax japonicus*) [13], mud Loach (*Misgurnus anguillicaudatus*) [14] and large yellow croaker (*Larimichthys crocea*) [15]. Despite the economic significance of Japanese flounder, genomic and transcriptomic data of this species remains scarce. Presently, only 16,000 expressed sequence tags (ESTs) and 5000 nucleotide sequences of flounder are deposited in the NCBI GenBank, still fewer entries compared to the other fish species mentioned above. The limited genomic sequences of flounder are far from sufficient to support the establishment of disease-resistant breeds or the development of effective vaccines and anti-microbial drugs against diseases.

In this study, we first employed the Illumina Miseq sequencing platform to sequence the spleen transcriptome of *P. olivaceus* and performed *de novo* assembly. Blastx searches against the NCBI non-redundant (nr) protein database annotated 21,391 sequences, which were subjected to three databases, including Gene Ontology (GO), Cluster of Orthologous Groups (COG) and Kyoto Encyclopedia of Genes and Genomes (KEGG). Furthermore, putative simple sequence repeats (SSRs) and single nucleotide polymorphisms (SNPs) were identified. Finally, a global survey of immune-relevant genes was performed and several immune signaling pathways were annotated in detail. These data will provide a rich resource to discover and identify novel genes. The immune-relevant sequences, SSRs, and SNPs identified here will facilitate our understanding of the mechanisms involved in the immune response and the development of effective measures in disease control.

## Results and Discussion

### *De novo* Assembly and Analysis

**Fig 1:**
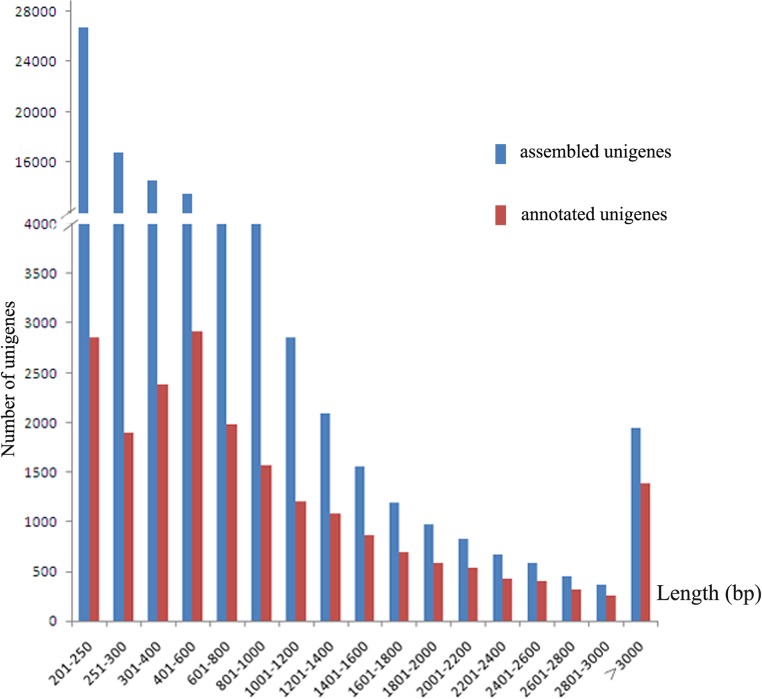
Length distribution of the assembled (blue) and the annotated unigenes (red) of *P. olivaceus*.

Table 1 summarizes statistics of flounder spleen transciptome using the Illumina Mi-Seq platform. A total of 14,699,453 raw reads, with an average read length of 251 bp were generated. After trimming the adaptor sequences and low-quality reads, 12,196,968 clean reads were generated and submitted to the NCBI Short Read Archive under accession number SRR1515192. Using Trinity software [16], the clean reads were assembled into 689,686 contigs with an average length of 166 bp. The contigs generated in this analysis were further assembled into 314,377 transcripts with a minimum length of 200 bp and an average read length of 363 bp using Trinity software. The transcripts were further assembled and clustered using the TIGR Gene Indices Clustering Tools (TGICL) with default parameters to reduce the data redundancy. The longest sequence in each cluster was retained and designated as a unigene. A total of 96,627 unigenes were assembled, with lengths ranging from 200 bp to 22,906 bp. The length distribution of the assembled unigenes is displayed in Fig. 1. Of the assembled unigenes, the majority of sequences (57,978, 60.22%) ranged from 200 to 400 bp, 20,336 (21.46%) from 400 to 800 bp, and 13,536 (14.01%) were longer than 1 kb. These data were comparable with those of previous studies [14, 17].

**Table 1:** Summary statistics of *P. olivaceus* transcriptome sequencing and assembly.

Description	Number
Before trimming	
Raw reads	14,699,453
Raw data(bp)	7,379,125,406
Average length(bp)	251
After trimming	
Clean reads	12,196,968
Clean data(bp)	4,660,132,029
Average read length(bp)	191
Assembly with Trinity software	
Contigs	689,686
Average length(bp)	166
Max read length(bp)	1,448
N50	166
Further assembly with Trinity software	
Transcripts (≥200bp)	314,377
Average length (bp)	363
Maximum length (bp)	22,906
N50 (bp)	692
Unigenes (≥200bp)	96,627
≥500bp	30,189

**Fig 2:**
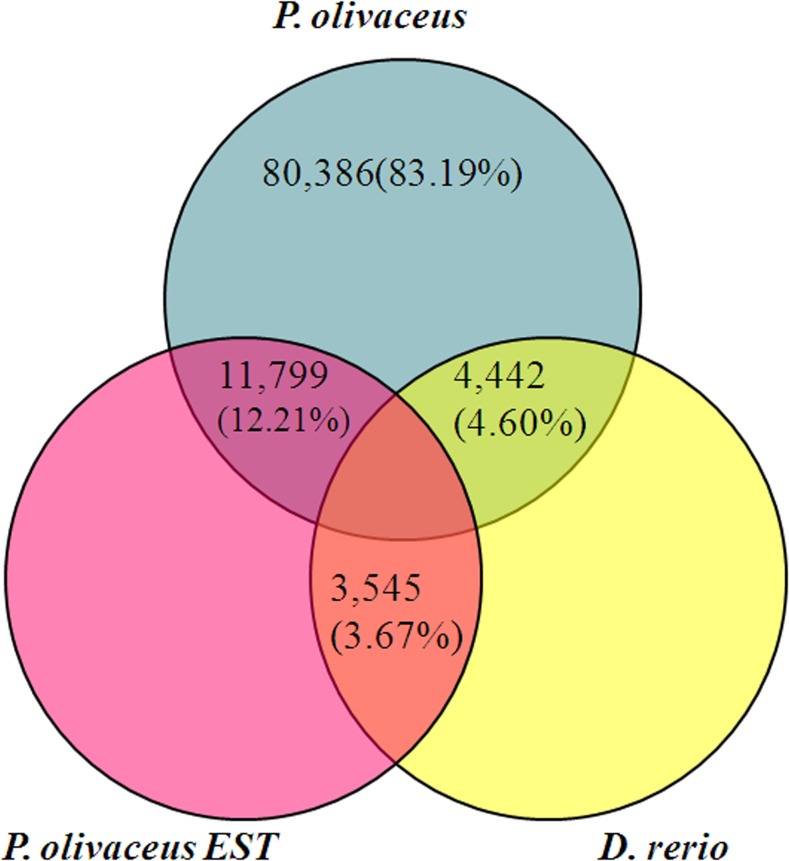
Venn diagram showing the comparison among *P. olivaceus* transcriptomic sequences with the known sequences from *D. rerio* and *P. olivaceus* EST deposited in the NCBI database.

To assess the abundance and coverage of the transcriptome data, the assembled unigenes were compared with known flounder EST and nucleotide sequences in the NCBI database. Before our study, there were 16,275 EST and 5,670 nucleotide sequences for *P. olivaceus* in the NCBI database. As shown in Fig. 2 as a Venn chart, 72.50% (11,799 out of 16,275) of the EST sequences could be matched in the transcriptomic unigenes, whereas only 12.21% (11,799 out of 96,627) of the transcriptomic sequences could be matched within the EST sequences of *P. olivaceus*. This result indicated that the flounder transcriptome covered a majority of the available EST sequences, providing abundant information besides available ESTs sequences.

To detect novel proteins, the flounder transcriptomic sequences were compared to unigenes from zebrafish (*D. rerio*) in the NCBI database (there are 53,558 zebrafish unigenes in the NCBI database, 2014-05-06). As shown in Fig. 2, only 4,442 (4.60%) sequences were shared between the flounder transcriptome and zebrafish unigenes, suggesting low homology between the two fish species. In total, 80,386 (83.19%) of the unigenes had no similarity to any protein identified within the known sequences of *P. olivaceus* and *D. rerio*, which could reflect a source of undiscovered new genes in other fish. Moreover, 3,545 (3.67%) sequences had homology with both *D. rerio* and *P. olivaceus* ESTs and probably represented well-conserved genes across these species.

### Annotation of assembled unigenes

To further elucidate the flounder transcriptome, the assembled 96,627 unigenes were annotated using the Nr (non-redundant protein) and SWISSPROT databases. There were a total of 21,391 (22.14%) unigenes with significant blast hits (E-value≤1E-5) to known proteins (Fig. 1, S1 Table). A considerable percentage of sequences (77.86%) did not retrieve good hits. The annotation was lower than those reported in high throughput sequencing studies conducted in other fish species, such as turbot (44.84%) [12] and mud loach (43.76%) [14], but comparable to that in crucian carp (17.44%) [18]. The low percentage of matched sequences might be due to many short reads obtained from sequencing, more than one-fourth of the unigene sequences were short (≤250 bp, 27.71%), which might hinder statistically significant matches. Among the annotated sequences, 7,786 (36.4%) of the sequences longer than 1000 bp had BLAST matches, whereas only 2,855 (13.3%) of the sequences shorter than 250 bp did, indicating that longer unigenes were more likely to obtain BLAST matches in the protein databases [14]. However, it is also possible that the flounder may have more unique genes compared to other species.

**Fig 3:**
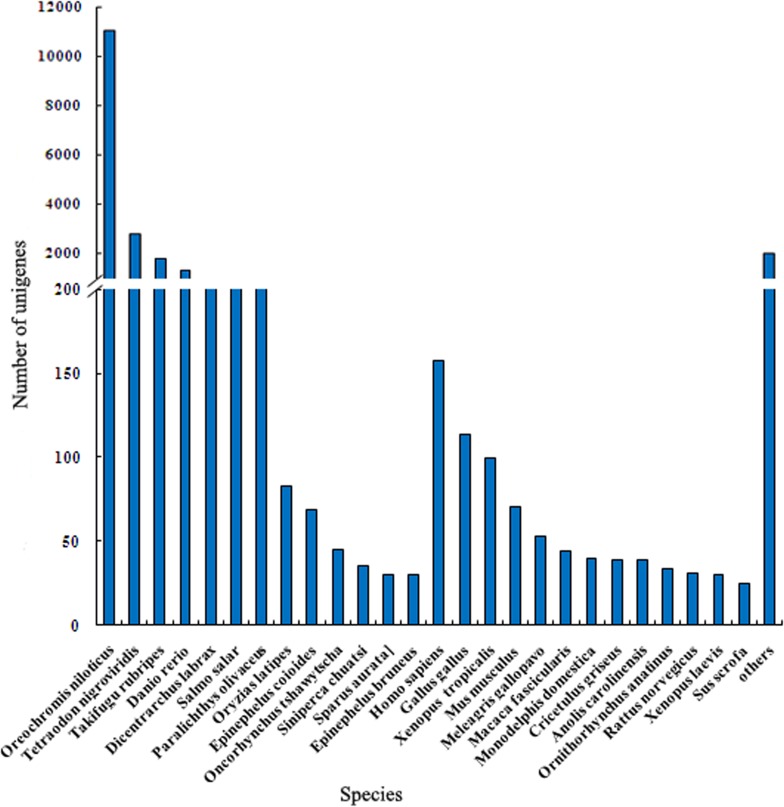
Distribution of top-hit species.

The species distribution of best hits is shown in Fig. 3. Among the BLAST hits, 18,627 (87.1%) originated from fish species, 881 (4.1%) from other vertebrates, including mammals, birds, amphibians, and reptiles, and 1,887 (8.8%) from other species, mainly plant and microorganisms. These data are similar to those found in other fish species [12, 14]. There were 13 fish species among the best hits and the species with the highest homology were *Oreochromis niloticus*, followed by *Tetraodon nigroviridis, Takifugu rubripes, Danio rerio, Dicentrarchus labrax*, and *Atlantic salmon*, all of which have known genome sequences. Flounder was the seventh and only 239 (1.1%) of the BLASTx top-hits matched *P. olivaceus* protein sequences. This result could be explained by the limited number of flounder proteins currently available in the NCBI database compared to other fish species. Overall, this work provided 96,627 unigenes for further genomic and transcriptomic studies in *P. olivaceus*. The vertebrate species with known genome sequences appearing in the top-hits included the mammal *Homo sapiens*, the chicken *Gallus fallus*, and the amphibian *Xenopus tropicals*. Some sequences showed homology to proteins from fish bacterial pathogens, including *Vibrio* sp., *Pseudomonas* sp. and *Edwardsiella* sp. These annotated sequences from microorganisms could be explained by the normal microbiota present in flounder tissues [14, 18].

### GO, COG and KEGG classification

**Fig 4:**
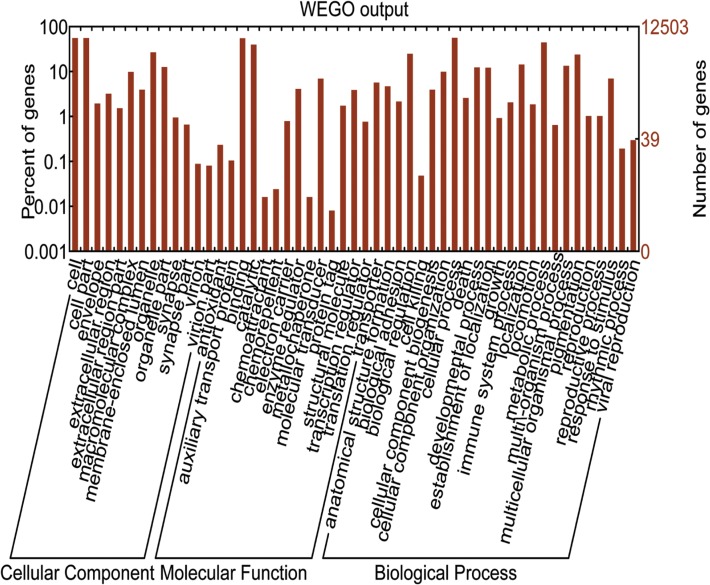
Gene ontology (GO) annotations of the annotated unigenes. 12,503 unigenes were assigned to three GO categories containing 52 functional subcategories.

Gene ontology (GO) is commonly used to categorize gene products and standardize their representation across species [19]. As shown in Fig. 4, 12,503 of the 21,391 unigenes annotated in the Nr and Swiss-Prot databases were classified into 52 functional subcategories under three GO terms corresponding to “biological process”, “molecular function,” and “cellular component” (S2 Table). Each unigene could be assigned to more than one GO term and altogether 31,356 unigenes were assigned to “biological process”, followed by “cellular component” (21,641 unigenes), and molecular function (14,794 unigenes). In the “biological process” category, the dominant subcategories were “cellular process” (6,995 unigenes, 55.90%), followed by metabolic process (5,525, 44.20%) and biological regulation (3,086, 24.70%). It is worth noting that a large number of immune-related sequences were enriched under “immune system process” (256, 2%) and “response to stimulus” (866, 6.90%). In the “cellular component” category, the most abundant terms were “cell” (6,896, 55.20%), “cell part” (6,896, 55.20%), “organelle” (3,339, 26.70%) and “organelle part” (1,567, 12.50%). Among the molecular function ontology, the predominant subcategories were “binding” (6,823, 54.60%) and “catalytic activity” (4,950, 39.60%).

**Fig 5:**
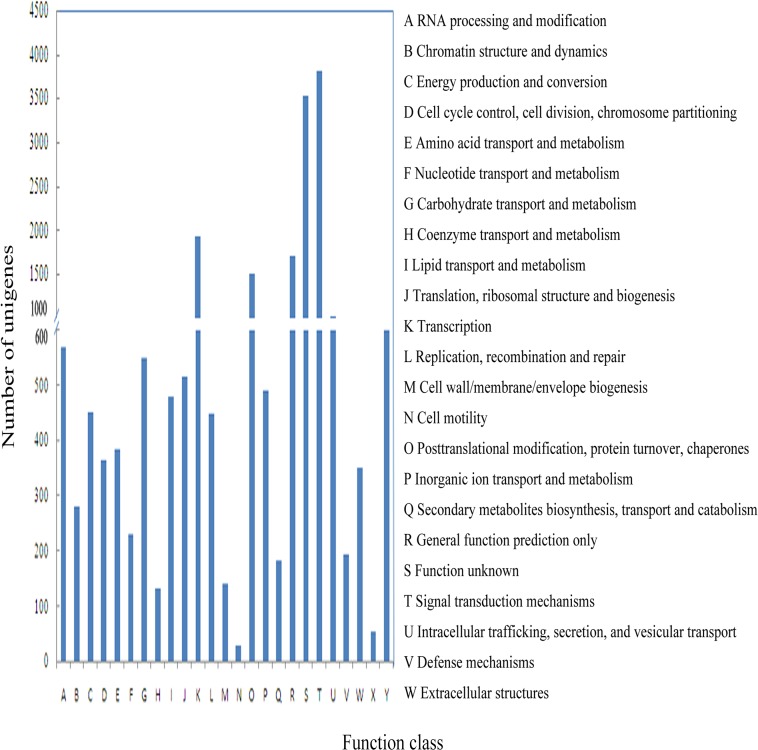
Cluster of Orthologous Groups (COG) annotations of annotated unigenes. 19,547 unigenes were classified into 26 COG categories.

The annotated unigenes were further grouped using the Cluster of Orthologous Groups (COG) database. As shown in Fig. 5, 19,547 unigenes were classified into 26 functional families (S3 Table). The predominant subfamily was “signal transduction mechanisms” (3,551, 19.11%), followed by “transcription” (1,828, 9.84%), “general function prediction only” (1,600, 8.61%), and “posttranslational modification protein turnover, chaperones” (1,435, 7.72%). A number of unigenes (168, 0.92%) were assigned to defense mechanisms that might be closely related to the flounder immune defense.

The Kyoto Encyclopedia of Genes and Genomes (KEGG) is a pathway database that records networks of molecular interactions among cells specific to particular organisms, enabling further analysis into the biological functions of genes. Using the KAAS [20], a total of 10,649 (49.78%) (S4 Table) assembled unigenes were assigned to 291 KEGG pathways corresponding to six categories: “Metabolism” (3,559, 31.54%), “Genetic information processing” (1,849, 17.36%), “Environmental information process” (2,379, 22.34%), “Cellular processes” (2541, 23.86%), “Organismal systems” (5,228, 49.09%), and “Human diseases” (6,676, 62.69%) (S5 Table). Among the identified functional categories, the “Infectious Diseases” cluster represented the largest group (2,772, 26.03%), followed by “Cancers” (2,189, 20.56%), “Signal Transduction” (1,774, 16.66%), “Immune System” (1,563, 14.68%), and “Nervous System” (1,229, 11.54%).

### Immune pathways annotation

Although immune-related genes in Japanese flounder have been previously characterized from ESTs or EST-based microarray chips [5, 21], studies on the immune system of this species are limited due to the lack of transcriptomic and genomic resources. To obtain a better overview of the immune system in flounder, we further identified immune-relevant unigenes from the transcriptome. A total of 1563 unigenes were homologous to immune-relevant genes based on the KEGG annotation and were classified into 15 functional pathways (Table 2), including “chemokine signaling” (196 unigenes, 12.54%), “T cell receptor signaling” (164, 10.49%) and “Leukocyte transendothelial migration pathway” (163, 10.43%). However, “Fc gamma R-mediated phagocytosis” (48/54, 88.89%), “B cell receptor signaling pathway” (44/54, 81.48%), “Leukocyte transendothelial migration” (55/73, 75.34%), and “Fc epsilon RI signaling pathway” (31/44, 70.45%) contained the highest ratios of identified genes versus the total number of known genes in the reference pathway. Approximately 83 immune-related genes were identified in a previous study [5], far less than the 1563 genes obtained in this transcriptome. We further described the main immune pathways identified in this flounder transcriptome.

**Table 2:** Immune pathways annotated in the *P.olivaceus* spleen transcriptome.

Signaling pathways	KO identifier	No. of unigenes	Mapped genes	Known genes
Hematopoietic cell lineage	ko04640	68	35	78
Complement and coagulation cascades	ko04610	65	42	69
Toll-like receptor signaling pathway	ko04620	110	53	76
NOD-like receptor signaling pathway	ko04621	58	28	51
RIG-I-like receptor signaling pathway	ko04622	62	37	53
Natural killer cell mediated cytotoxicity	ko04650	122	44	79
Antigen processing and presentation	ko04612	61	26	41
T cell receptor signaling pathway	ko04660	164	56	83
B cell receptor signaling pathway	ko04662	116	44	54
Fc epsilon RI signaling pathway	ko04664	85	31	44
Fc gamma R-mediated phagocytosis	ko04666	155	48	54
Leukocyte transendothelial migration	ko04670	163	55	73
Intestinal immune network for IgA production	ko04672	26	14	37
Chemokine signaling pathway	ko04062	196	87	143
Cell adhesion molecules (CAMs)	ko04514	112	59	99

**Toll-like receptor signaling pathway.** Toll-like receptors (TLRs) are responsible for the recognition of specific pathogen-associated molecular patterns, and consequently activate signal pathways leading to inflammatory and interferon responses through a MyD88-dependent pathway or a MyD88-independent pathway [22]. In flounder, approximately 17 TLR-relevant immune genes were characterized [5], including TLR3, TLR5, TLR 9, TLR 14, interferon regulatory 5, and myeloid differentiation primary response protein (MyD88). In this study, 56 unigenes mapped to the TLR signaling pathway consisting of 76 known genes (S1 Fig., S6 Table). Similar to some fish species like *Fugu* and *Tetraodon* [23, 24], flounder appears to lack TLR4, TLR6, and TLR10, as well as the co-stimulatory molecules MD2 and CD14 as they were undetected in our flounder transcriptome, as well as cDNA libraries and EST-based microarray DNA chips [5]. In addition, many important molecules in the TLR signaling pathway were identified for the first time in flounder, including toll-interacting protein (TOLLIP), the interleukin-1 receptor-associated kinases (IRAK1 and IRAK4), inhibitor of nuclear factor kappa-B kinases (IKKα, IKKβ, IKKγ), and TNF receptor-associated factor (TRAF3, TRAF6). Among these identified molecules, TOLLIP and IRAKs are of particular interest due to their important roles as regulators of NF-kB signaling in mammals [25, 26]. In the teleost, few studies have reported the exact roles of these two molecules. Presently, TOLLIP-encoding genes in Atlantic salmon (*Salmo salar*) [27] and an IRAK-1 cDNA sequence in mandarin fish [28] were cloned, and in grass carp (*Ctenopharyngodon idellus*), the interaction of Tollip with IRAK-1 proteins was demonstrated to be part of the TLR signaling pathway [29]. The identification of new genes, such as TOLLIP and IRAKs, involved in the TLR signaling pathway in flounder can provide new insights into the role of the TLR signaling pathway in teleosts and will be a subject of our future studies.

**Complement and coagulation pathway.** Consisting of more than 30 proteins, the complement system defends against infectious microorganisms through the innate immune system and antibody-mediated immunity [30]. In the flounder, several important components in the complement pathway have been detected from cDNA libraries and EST-based microarray DNA chips [5], including complement components (C1, C3, C4, C5, C7, C8, C9), complement factors (Bf, Hf), and the antigen CD59 [6, 21]. Only two of them, C8b and C9, were cloned and characterized [31]. In this study, 18 unigenes were mapped to the complement signaling pathway that consists of 27 known genes (S2 Fig., S7 Table). Some molecules were identified for the first time in the flounder, such as C2, C6, C8g, C5a anaphylatoxin chemotactic receptor (C5AR1), mannan-binding lectin serine protease 2 (MASP2), C1 inhibitor (C1INH), decay accelerating factor (DAF), and complement factors H (Hf1).

As part of the homeostatic process and thrombosis, the coagulation system is also a part of the innate immune system that responds to infection at an early stage [32]. In the present study, we identified the main putative central molecules in the coagulation cascades, such as coagulation factors (F2, F3, F5, F7, F8, F10 and F13), coagulation factor II (thrombin) receptor (F2R), coagulation factor XIII A1 polypeptide (F13A1), bradykinin receptor (B2), and plasminogen (PLG). Only one of them, fibrinogen B, was previously reported in the flounder [33].

Both the complement and coagulation systems belong to the “first line of defense” after injury and exposure to microbial intruders [34]. Interactions between complement and coagulation have been investigated in mammals and shown to be involved in inflammatory pathogenesis [35]. However, no study has reported on the interplay of complement and coagulation in fish. The elevated number of complement and coagulation components detected in the flounder will thus enable the characterization of mechanisms that respond to bacterial infection.

**B cell and T cell receptor signaling pathway.** Both B lymphocytes and T lymphocytes participate in specific antigen defense. B cells are involved in adaptive humoral immunity through antibody production, antigen presentation, and memory B cells development after antigen-mediated activation. B cell activation is achieved through the binding of antigen to the B cell receptor (BCR) located on the outer surface of B cells [36]. Several sequences homologous to immunoglobulin L, IgD, and IgM were previously detected in the flounder [5]. In this transcriptome study, 44 unigenes were mapped to the B cell signaling pathway that consists of 54 known genes (S3 Fig., S8 Table), such as clusters of differentiation antigens (CD22, CD79A, CD81), phosphatidylinositol-3, 4, 5-trisphosphate 5-phosphatase 1 (SHP1), tyrosine-protein kinase (LYN), RAC serine/threonine-protein kinase (AKT), Ras-related C3 botulinum toxin substrate 1 (Rac1), RAS guanyl-releasing protein (RasGRP3) and mucosa-associated lymphoid tissue lymphoma translocation protein 1 (MALT1).

T cells are involved in cell-mediated immunity through phagocyte activation, antigen-specific cytotoxic T-lymphocytes, and the release of various cytokines in response to an antigen. T cell activation is achieved when the T cell receptor (TCR) recognizes antigens presented by major histocompatibility complex (MHC) molecules [37]. In this study, 56 unigenes were mapped to the T cell signaling pathway that consists of 83 known genes (S4 Fig., S9 Table). Some of these unigenes have been previously identified [5, 6], such as genes encoding TCR α, β, γ and δ, CD3, CD3ε, CD4 and CD8. Other genes were discovered for the first time in the flounder, such as lymphocyte-specific protein tyrosine kinase (LCK), zeta-chain associated protein kinase (ZAP-70), SLP-76 in the PLC-γ1 pathway, PI3K, AKT, COT in the PI3K pathway, and RasGRP1 and MEK1/2 in the Ras-MAPK pathway. LCK and ZAP-70 are particularly interesting, as studies in fish relevant to both molecules are limited. As known from mammalia, LCK phosphorylates downstream molecules like ZAP-70 after binding to CD4 or CD8, and ZAP-70 plays a critical role in T-cell signaling and B-cell development [38]. LCK and ZAP-70 have only been previously characterized in Atlantic halibut (*Hippoglossus hippoglossus* L.) [39, 40]. Therefore, B cell receptor and T cell receptor signaling pathway members detected in the flounder transcriptome will enable investigations into the mechanisms in this pathway.

**Cytokines.** Cytokines are a group of proteins responsible for the communication of immune system cells, hematopoietic cells, and other cell types [41]. In this study, we detected unigenes of major cytokines, including interleukins (ILs) (seven unigenes), interferons and interferon receptors (eight unigenes), tumor necrosis factors (eight unigenes), chemokines (20 unigenes), and colony stimulating factors (five unigenes). Chemokines are classified into four main subfamilies in mammals: CXC, CC, CX3C, and XC [42]. Among teleost fish, C chemokines have only been identified in zebrafish, whereas CX3C chemokines have never been reported [43]. In the flounder, several chemokine-associated sequences have been previously reported [5, 6], including CXC13, CCL3, and MCP1. In this flounder transcriptome study, we identified sequences corresponding to CC and CXC chemokines and chemokine receptors (S10 Table), some identified for the first time in the flounder, including CXCL12, CXCL14, CCL20, CCL25, XCR1, CCR7, CXCR3, CXCR4, and CXCR5.

ILs function in cell proliferation, maturation, migration, and adhesion, immune cell differentiation, and immune cell activation [44]. Several IL-associated members have been previously reported in the flounder, including IL-1β, IL-6, IL-8, IL11b, and IL-1RII [5, 6]. Our transcriptome study is the first to detect other ILs and IL receptors, including IL1R2, IL6R, IL7R, and IL11RA. For tumor necrosis factors (TNFs) essential for inflammation, host defense, autoimmunity, organogenesis, cellular apoptosis, and differentiation [45], four members of this family have been identified in the flounder, including TNFR1, TNFR2, TNF-a, FAS ligand, and CD40 [5, 6]. In this study, we identified other TNF-associated molecules for the first time, including TNSF5, TNSF10, and TNSF13B. Among the colony-stimulating factors (CSFs), which regulate intracellular signaling pathways that cause cell proliferation and differentiation into white blood cells [46], we detected two CFS-associated molecules: macrophage colony-stimulating factor 1 receptor (CSF1R) and granulocyte colony-stimulating factor receptor (CSF3R). These are the first CSF-associated molecules identified in the flounder.

### SSR and SNP discovery

SSRs and SNPs are efficient genetic tools to support the development of a linkage map or marker-assisted selection breeding programs in different species [47, 48]. A total of 40,928 putative SSR loci were discovered in 21,391 unigenes. Dinucleotide repeat motifs were the most common with a frequency of 42.17%, followed by mono- (37.68%), tri-(17.77%), tetra- (2.18%), penta-(0.14%) and hexanucleotide (0.06%) repeats (S11 Table). Moreover, a total of 47,362 putative SNPs were identified, including 28,142 transitions and 19,220 transversions. The proportions of transition substitutions were 29.93% for C/T and 29.49% for A/G, compared with smaller proportions of transversions for A/C (10.07%), G/T (10.51%), A/T (10.33%) and C/G (9.67%) (S12 Table). Furthermore, immune-relevant unigenes were analyzed to identify putative SNPs. Among the 1,563 immune unigenes, a total of putative 1,579 SNPs were detected, which were distributed in 339 unigene sequences (S13 Table). Of the 339 unigenes, 220 had at least more than two putative SNPs loci. Some important immune-relevant unigenes had more than 10 SNP loci, including MHC I (61), NOD (21), CD44 (18), TLR2 (12), and IRAK1 (11). MHC is considered a candidate molecular marker of an association between polymorphism and resistance/susceptibility to diseases [49]. In flounder, the link between alleles of MHC class II genes and disease resistance or susceptibility to bacterial infection has been demonstrated [11]. Taken together, the putative SSRs and SNPs found in the flounder transcriptome can provide potential genetic markers for applications in population genetics, comparative genomics, as well as marker-assisted selection breeding program.

### QRT-PCR analysis of unigenes

**Fig 6:**
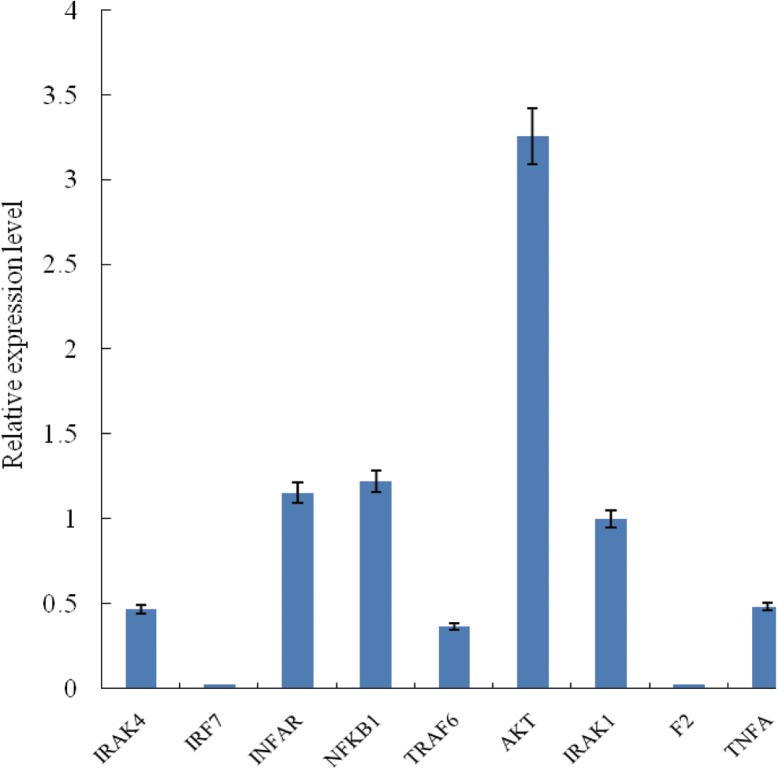
Quantitative Real-time PCR (qRT—PCR) validation of the expressed genes in transcriptome sequencing. IRAK4: interleukin-1 receptor-associated kinase 4; IRF7: interferon regulatory factor 7; INFAR: interferon receptor 1; NFKB1: nuclear factor NF-kappa-B p105 subunit; TRAF6: TNF receptor-associated factor 6; AKT: RAC serine/threonine-protein kinase; IRAK1: interleukin-1 receptor-associated kinase 1; F2: coagulation factorII; TNFA: tumor necrosis factor superfamily. Values are presented as means ± standard deviation（n = 5）and the error bars indicate the standard deviation.

To validate the assembled unigenes, 10 unigenes related to immune signaling pathways were selected for qRT-PCR (quantitative real–time PCR) (S14 Table). As shown in Fig. 6, the tested unigenes displayed different expression levels, AKT showed the highest expression level (followed by NFKB1, INFAR and IRAK1), while IRF7 and F2 had the lowest expression levels. This result was consistent with the Illumina sequencing data, suggesting the reliability and accuracy of this transcriptome analysis.

## Conclusion

This study investigated the transcriptome profile from the spleen of *P. olivaceus* using the Illumina paired-end RNA sequencing. To the best of our knowledge, this is the first *de novo* sequencing and transcriptome assembly for *P. olivaceus*, which lacks a reference genome. This abundant sequence resource provides a strong basis for future genomic research on flatfish and supports the in-depth annotation of the vertebrate genome. Globally identified immune-relevant genes and putative immune signaling pathways in *P. olivaceus* provide valuable information for further investigations into the immune response and disease prevention of fish. The detected SSRs and SNPs are important data for development of a linkage map or marker assisted breeding programs for flounder.

## Materials and Methods

### Ethics Statement

This experiment as described was carried out in strict accordance with the approval of the Animal Care and Use Committee of the Institute of Oceanology, Chinese Academy of Sciences.

### Fish

Japanese flounder (mean weight of 200 g) were purchased from a local fish farm (Jiaonan, Qingdao, China). The fish were maintained in aerated seawater tanks at 20 ± 2°C with a flow-through water system. After 2 weeks of acclimation, fish were randomly sampled to detect bacteria from the liver, spleen, blood, and kidney. Only bacteria-free fish were used for experimentation. After anesthesia using MS222, 20 fish were randomly sacrificed. Spleens were collected and flash frozen in liquid nitrogen and stored at -80°C.

### Total RNA extraction and sample preparation for RNA-Seq

Total RNA was extracted from the spleen using the Total RNA KIT II, (Omega, USA) following the manufacturer’s protocol. All RNA was treated with RNase free DNase I provided by the kit. The quantity, purity and integrity of RNA were measured on a 1.2% (w/v) agarose gel and on a Nanodrop-1000 spectrophotometer. Samples with higher quality (absorbance ratios at 260 nm/280 nm>1.9) were selected for high-throughput sequencing. The extracted total RNA was resuspended in distilled water and stored at -80°C until use. After enrichment using oligo-dT-attached magnetic beads, the purified mRNA was fragmented using divalent cations under elevated temperature, and then applied as a template for first-strand cDNA synthesis using random primers and reverse transcriptase. The second-strand cDNA was synthesized using RNase H (Invitrogen, USA) and DNA polymerase I. The library was constructed following the manufacturer’s protocol.

### Assembly, comparative analysis, and functional annotation of the transcriptome

Transcriptome sequencing was conducted using the Illumina Mi-Seq sequencing platform. To better assemble the entire transcriptome *de novo*, a paired-end (PE) sequencing strategy was used. Raw PE reads with an average length of 251 bp were generated. All sequences were examined for possible sequencing errors. Adaptor sequences and low quality sequences were trimmed. Short sequences (< 50 bp) were removed using a custom Perl program [50]. The resulting high-quality sequences were deposited in the NCBI database and *de novo* assembled into contigs and transcripts with Trinity software, as described for *de novo* transcriptome assembly without a reference genome [16]. To reduce data redundancy, transcripts with a minimum length of 200 bp were assembled and clustered using TGICL under default parameters [51]. The longest sequences in each cluster were reserved and designated as unigenes. The assembled unigenes of the flounder were compared to the ESTs of *P. olivaceus* and unique nucleotide sequences of *D. rerio* deposited in the NCBI databases using the BLASTn algorithm. There were 53,558 unique nucleotide sequences for *D. rerio*, and 16,275 ESTs and 5670 nucleotide sequences for *P. olivaceus*, respectively. The E-value cut-off was 1E-5. Sequence homology searches were performed using local BLASTX programs against sequences in the NCBI non-redundant (nr) protein database and the SWISS-PROT database (E-value ≤ 1E-5) [52]. Unigenes were tentatively identified according to top hits against known sequences. The resulting unigenes were used as references for the determination of the GO term and COG term, and were analyzed further using KEGG.

### Identification of SSR and SNP markers

MicroSAtellite (http://pgrc.ipk-gatersleben.de/misa/) was used to identify putative SSRs in the unigenes from the assembled transcirpt. The parameters were adjusted in order to identify perfect mono-, di-, tri-, tetra-, penta- and hexanucleotide motifs with a minimum of 10, 6, 5, 5, 5, and 5 repeats, respectively. To identify putative SNP in the transcriptome of Japanese flounder, QualitySNP was used to analysis SNP loci from the 314,377 transcripts [53]. SNP identification was limited to the transcripts containing at least four reads for each allele and required the minor allele frequency 25%.

### Gene expression validation

To verify the quality of sequences assembled in this study, 10 unigenes homologous to known proteins were validated by qRT-PCR. QRT-PCR was performed using the Bio-RAD CFX connect Real-Time System (Applied Biosystems, USA) with SYBR Green (Invitrogen, USA) as the fluorescent dye according to the manufacturer’ s protocols. First-strand cDNA was synthesized from 2 μg of total RNA with reverse transcriptase (Takara, Japan) and random primers, and the resulting products were used as a template for qRT-PCR. Primers were designed with Primer Premier 5 according to unigenes. The specific primers used for qRT-PCR are listed in S14 Table. The qRT-PCR thermal cycling condition for all reactions was 95°C for 1 min 50 s, followed by 40 cycles of 95°C for 10 s, and 61°C for 33 s. All reactions were performed in biological triplicates, and the results are expressed relative to the expression levels of β-actin in each sample using the 2^-ΔΔCT^ method [54]. Each sample was first normalized to the amount of added template by comparison with the abundance of β-actin mRNA.

## Supporting Information

S1 Fig A KEGG map of Toll-like Receptor (TLR) signaling pathway populated with unigenens coding for corresponding molecules. Proteins identified from the founder transcriptome were shown in red and absent proteins in blue.
(TIF)

S2 Fig A KEGG map of complement and coagulation cascades signaling pathway populated with unigenens coding for corresponding molecules. Proteins identified from the flounder transcriptome are in red and absent proteins are in blue.
(TIF)

S3 Fig A KEGG map of B cell receptor signaling pathway. Proteins identified from the flounder transcriptome are shown in red and absent proteins in blue.
(TIF)

S4 Fig A KEGG map of T cell receptor signaling pathway. Proteins identified from the flounder transcriptome are shown in red and absent proteins in blue.
(TIF)

S1 Table Annotation information of *P. olivaceus* spleen transcriptome. (XLS)

S2 Table Detailed information of GO pathway analysis. (XLS)

S3 Table Detailed information of COG pathway analysis. (XLS)

S4 Table Detailed information of KEGG pathway analysis. (XLS)

S5 Table KEGG classification of the annotated unigenes. (DOC)

S6 Table Information of the unigenes identified as homologous to molecules involved in the Toll-like receptor signaling pathway. (XLS)

S7 Table Information of the unigenes identified as homologous to molecules involved in the Complement and coagulation cascades. (XLS)

S8 Table Information of the unigenes identified as homologous to molecules involved in the B cell receptor signaling pathway. (XLS)

S9 Table Information of the unigenes identified as homologous to molecules involved in the T cell receptor signaling pathway. (XLS)

S10 Table Information of the unigenes identified as homologous to molecules involved in the chemokine signaling pathway. (XLS)

S11 Table Statistics of SSR identified from Japanese flounder transcriptome. (DOC)

S12 Table Number of each type of SNPs detected from Japanese flounder transcriptome. (DOC)

S13 Table Number of SNP of immune unigenes identified in the transcriptome of Japanese flounder. (XLSX)

S14 Table Genes and specific primers used for quantitative real-time PCR. (DOC)
