# Correction: *De Novo* Assembly of the Japanese Flounder (*Paralichthys olivaceus*) Spleen Transcriptome to Identify Putative Genes Involved in Immunity

**DOI:** 10.1371/journal.pone.0131146

**Published:** 2015-06-17

**Authors:** Lin Huang, Guiyang Li, Zhaolan Mo, Peng Xiao, Jie Li, Jie Huang

There are errors in the Funding section. The correct funding information is as follows: This research was supported by the Hi-tech Research and Development Program of China (Grant No. 2012AA10A408 and 2012AA092203), the National Natural Sciences Foundation of China (Grant No. 31072245 and 31372567), the special foundation under the Construction Programme for ‘Taishan Scholarship’ of Shandong Province of China, and Special Scientific Research Funds for Central Non-profit Institute, Yellow Sea Fisheries Research Institute (20603022013010). The funders had no role in study design, data collection and analysis, decision to publish, or preparation of the manuscript.
