# Supplementary figures and images for: De Novo Assembly of the Japanese Flounder (Paralichthys olivaceus) Spleen Transcriptome to Identify Putative Genes Involved in Immunity
Source: PLoS One. 2015 Feb 27;10(2):e0117642. doi: 10.1371/journal.pone.0117642 (PMC4344349; doi:10.1371/journal.pone.0117642)

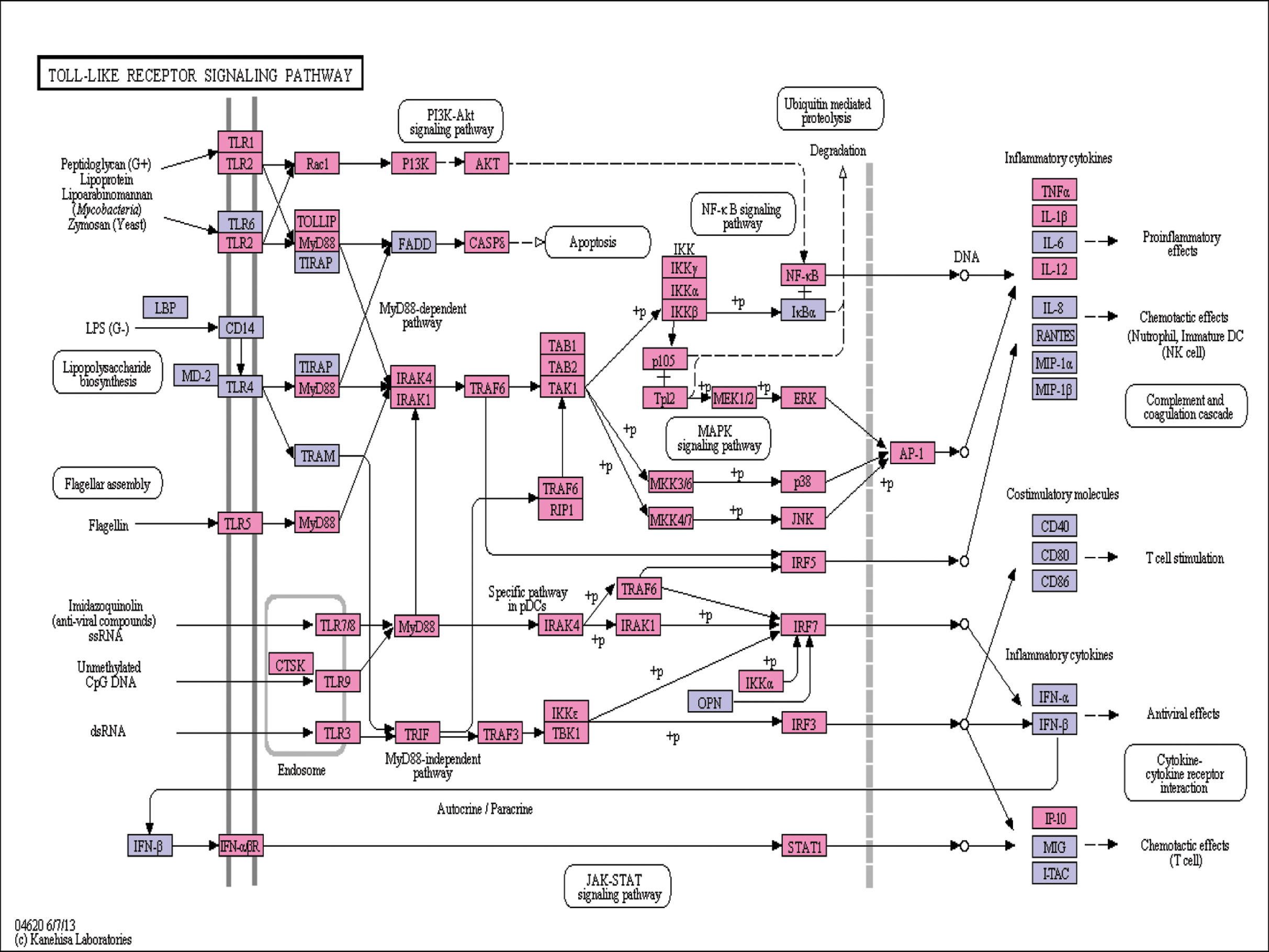

Supplement: S1 Fig — Proteins identified from the founder transcriptome were shown in red and absent proteins in blue. (TIF) [file pone.0117642.s001.tif]

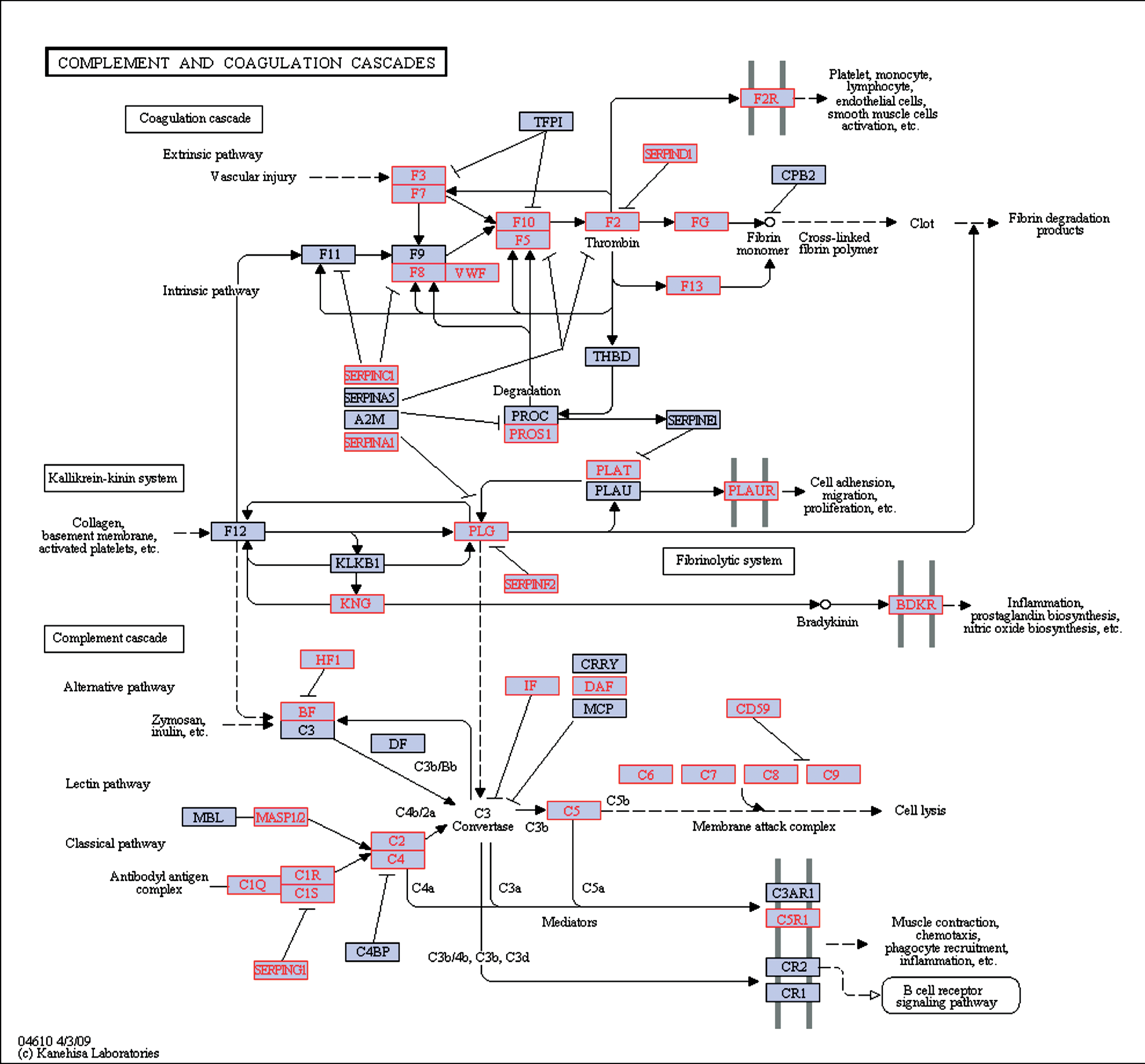

Supplement: S2 Fig — Proteins identified from the flounder transcriptome are in red and absent proteins are in blue. (TIF) [file pone.0117642.s002.tif]

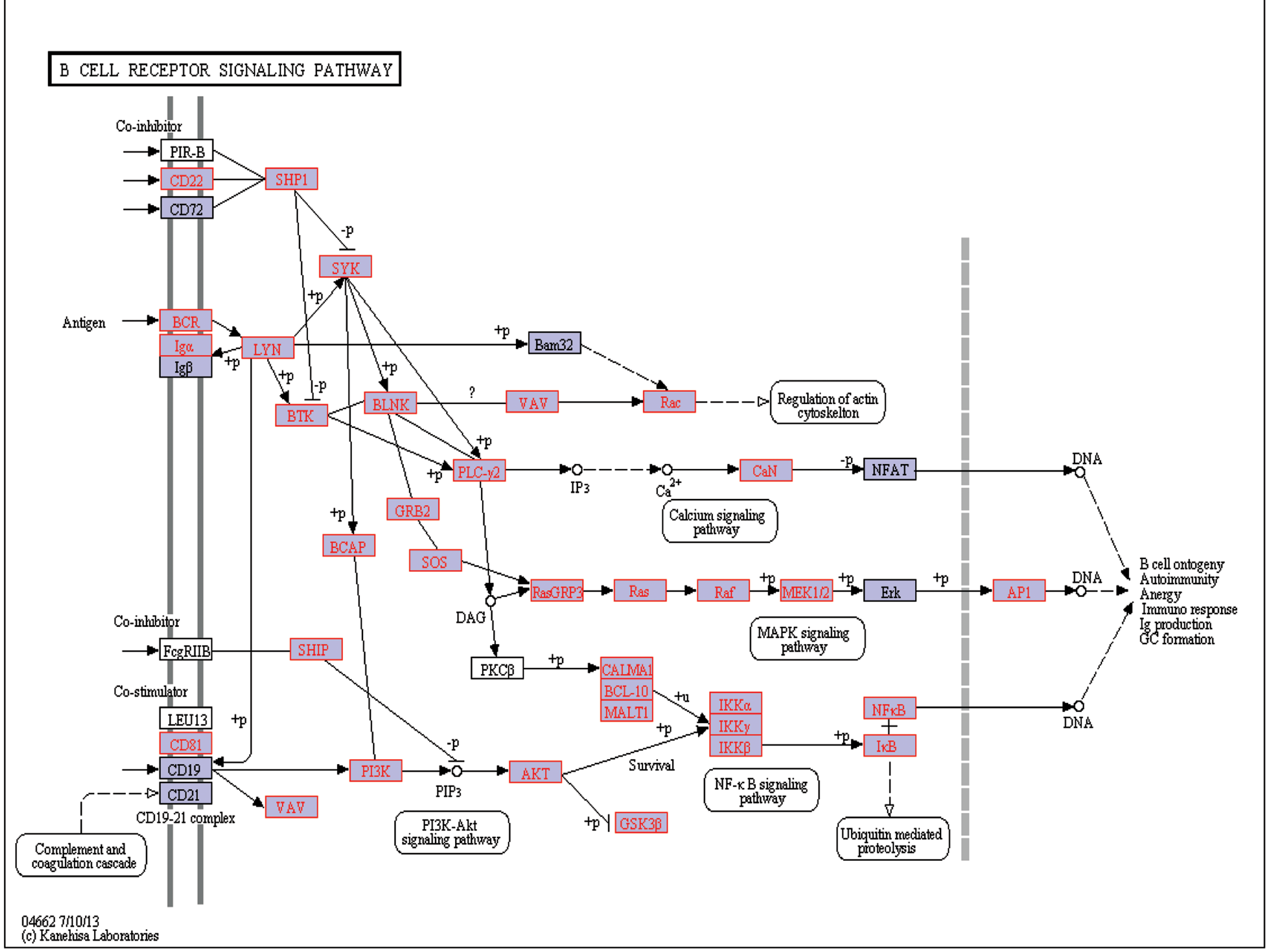

Supplement: S3 Fig — Proteins identified from the flounder transcriptome are shown in red and absent proteins in blue. (TIF) [file pone.0117642.s003.tif]

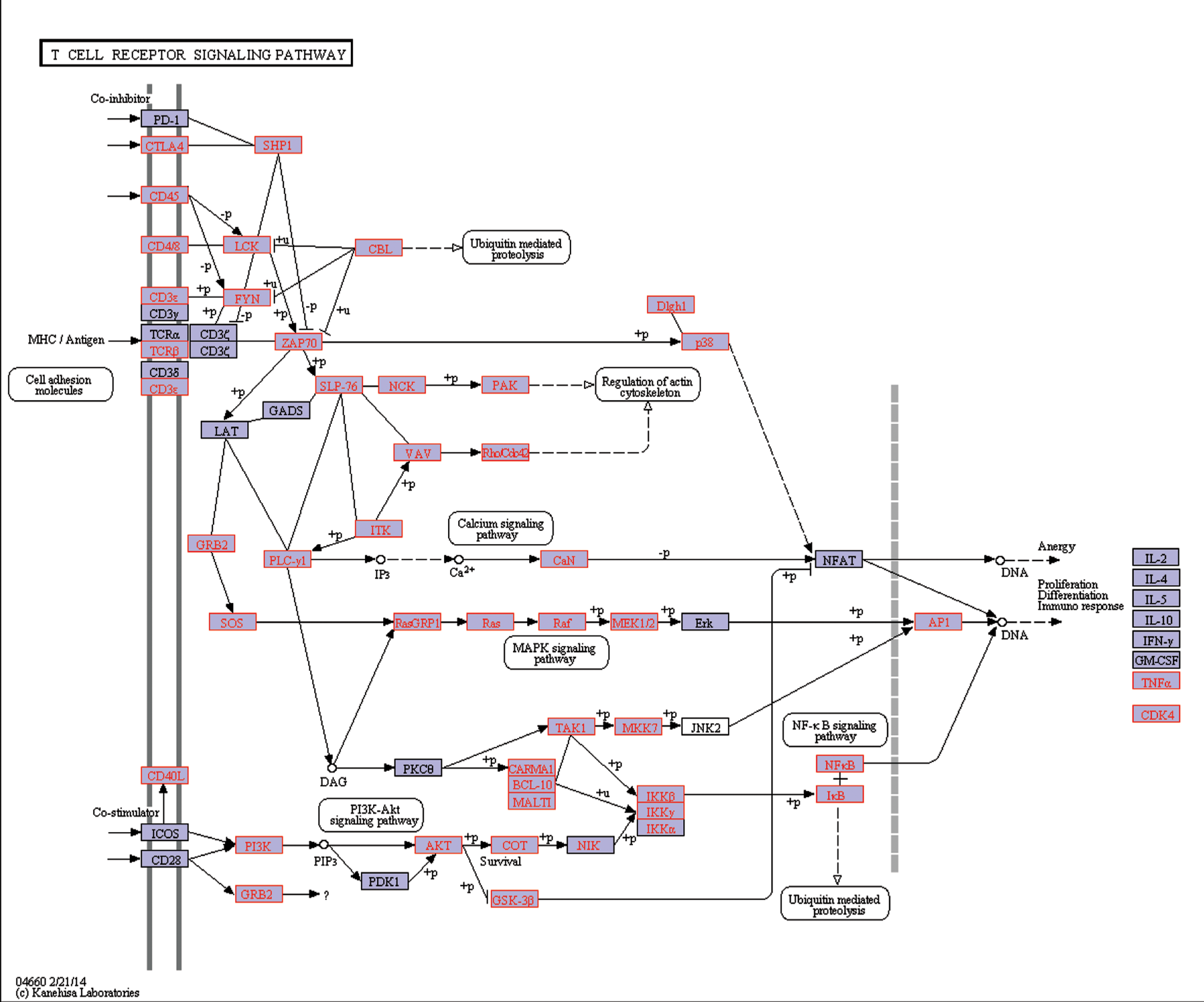

Supplement: S4 Fig — Proteins identified from the flounder transcriptome are shown in red and absent proteins in blue. (TIF) [file pone.0117642.s004.tif]
