# Supplementary material for: De Novo Assembly of the Japanese Flounder (Paralichthys olivaceus) Spleen Transcriptome to Identify Putative Genes Involved in Immunity
Source: PLoS One. 2015 Feb 27;10(2):e0117642. doi: 10.1371/journal.pone.0117642 (PMC4344349; doi:10.1371/journal.pone.0117642)
Supplement: S5 Table — (DOC) [file pone.0117642.s009.doc]

Table S5 **KEGG classification of the annotated unigenes**

| **KEGG category** | **No. of unigenes** |
| --- | --- |
| **Metabolism** |  |
| Carbohydrate Metabolism | 781 |
| Energy Metabolism | 343 |
| Lipid Metabolism | 509 |
| Nucleotide Metabolism | 426 |
| Amino Acid Metabolism | 541 |
| Metabolism of Other Amino Acids | 154 |
| Glycan Biosynthesis and Metabolism | 373 |
| Metabolism of Cofactors and Vitamins | 203 |
| Metabolism of Terpenoids and Polyketides | 31 |
| Biosynthesis of Other Secondary Metabolites | 55 |
| Xenobiotics Biodegradation and Metabolism | 143 |
| **Genetic Information Processing** |  |
| Transcription | 263 |
| Translation | 541 |
| Folding, Sorting and Degradation | 669 |
| Replication and Repair | 376 |
| **Environmental Information Process** |  |
| Membrane Transport | 85 |
| Signal Transduction | 1,774 |
| Signaling Molecules and Interaction | 520 |
| **Cellular Processes** |  |
| Transport and Catabolism | 762 |
| Cell Motility | 300 |
| Cell Growth and Death | 700 |
| Cell Communication | 779 |
| **Organismal Systems** |  |
| Immune System | 1,563 |
| Endocrine System | 745 |
| Circulatory System | 226 |
| Digestive System | 615 |
| Excretory System | 260 |
| Nervous System | 1,229 |
| Sensory System | 109 |
| Development | 404 |
| Environmental Adaptation | 77 |
| **Human Diseases** |  |
| Cancers | 2,189 |
| Immune Diseases | 231 |
| Neurodegenerative Diseases | 702 |
| Substance Dependence | 351 |
| Cardiovascular Diseases | 324 |
| Endocrine and Metabolic Diseases | 107 |
| Infectious Diseases | 2,772 |
