# Supplementary material for: De Novo Assembly of the Japanese Flounder (Paralichthys olivaceus) Spleen Transcriptome to Identify Putative Genes Involved in Immunity
Source: PLoS One. 2015 Feb 27;10(2):e0117642. doi: 10.1371/journal.pone.0117642 (PMC4344349; doi:10.1371/journal.pone.0117642)
Supplement: S11 Table — (DOC) [file pone.0117642.s015.doc]

**Table S11. Statistics of SSR identified from Japanese flounder transcriptome**

| Microsatellites identified |  |
| --- | --- |
| Mono-nucleotide | 15,422 |
| Di-nucleotide | 17,261 |
| Tri-nucleotide | 7,271 |
| Tetra-nucleotide | 891 |
| Penta-nucleotide | 57 |
| Hexa-nucleotide | 26 |
| Number of sequences containing more than 1 SSR | 7,226 |
