# Supplementary material for: De Novo Assembly of the Japanese Flounder (Paralichthys olivaceus) Spleen Transcriptome to Identify Putative Genes Involved in Immunity
Source: PLoS One. 2015 Feb 27;10(2):e0117642. doi: 10.1371/journal.pone.0117642 (PMC4344349; doi:10.1371/journal.pone.0117642)
Supplement: S12 Table — (DOC) [file pone.0117642.s016.doc]

**Table S12 Number of each type of SNPs detected from Japanese flounder transcriptome**

| SNP type | Transitions | | Transversions | | | |
| --- | --- | --- | --- | --- | --- | --- |
|  | A/G | C/T | A/T | G/T | C/G | A/C |
| Number of SNPs | 13,965 | 14,177 | 4,893 | 4,977 | 4,580 | 4,770 |
| Percentage | 29.49% | 29.93% | 10.33% | 10.51% | 9.67% | 10.07% |
