# Supplementary material for: De Novo Assembly of the Japanese Flounder (Paralichthys olivaceus) Spleen Transcriptome to Identify Putative Genes Involved in Immunity
Source: PLoS One. 2015 Feb 27;10(2):e0117642. doi: 10.1371/journal.pone.0117642 (PMC4344349; doi:10.1371/journal.pone.0117642)
Supplement: S14 Table — (DOC) [file pone.0117642.s018.doc]

**Table S14 Genes and specific primers used for qRT- PCR**

| Unigene ID | Gene | Description | Forward Primer(5’-3’) | Reverse Primer(5’-3’) |
| --- | --- | --- | --- | --- |
| gi|311294698 | β-actin | beta-actin | GACCTGACAGACTACCTCATG | AGTTGAAGGTGGTCTCGTG |
| Locus_1032_Transcript_5/6_Confidence_0.429_Length_2180 | IRAK1 | interleukin-1 receptor-associated kinase 1 | CGGTGTCTGTGAAGGAGGAGGAT | CGTGAATAAGCGGTGCGTGTCT |
| Locus_1_Transcript_210264/226691_Confidence_1.000_Length_475 | IRAK4 | interleukin-1 receptor-associated kinase 4 | AACAGAAGCAGCAGCCAGTAACC | GCGGTACTCACAGCAACACATCT |
| Locus_1_Transcript_110524/226691_Confidence_1.000_Length_1696 | C2 | complement  C2 | GCACGGCAAGCAGATAGGTCT | CGGCAGAGTGATGTCCAGCAA |
| Locus_1_Transcript_204450/226691_Confidence_1.000_Length_647 | INFAR1 | interferon receptor 1 | AGTACCACGTCCAAGCCTTTAG | CCAGCACCAACACCTCCAT |
| Locus_1_Transcript_140863/226691_Confidence_1.000_Length_1670 | NFKB1 | nuclear factor NF-kappa-B p105 subunit | GAGCGTCCAGCGAGAAGAACA | ACCAGACTGTGAGCGTGAAGC |
| Locus_17260_Transcript_1/1_Confidence_1.000_Length_722 | Traf6 | TNF receptor-associated factor 6 | CCTGCGTCACCAATCGTCCATC | GCTATCCTCCTCTAGTGGGCTCAG |
| Locus_1_Transcript_185435/226691_Confidence_1.000_Length_1387 | AKT | RAC serine/threonine-protein kinase | GTATGCTGGCAGAGTAGGAGAAC | CAGGTAACATCAGAGACAGACACA |
| Locus_1_Transcript_144083/226691_Confidence_1.000_Length_899 | IRF-7 | interferon regulatory factor 7 | GGTGGACCAGGAGACATGCAGTAA | GACAATCCCAGCAGCCCAGAAC |
| Locus_29264_Transcript_1/2_Confidence_0.667_Length_1069 | F2 | coagulation factorII | GAAAATCTGCACGGCAACAC | CATAAACAAATAAGAACGAGCGAC |
| Locus_1_Transcript_113498/226691_Confidence_1.000_Length_438 | TNFA | tumor necrosis factor superfamily | GTAGAGACGAGGAAGACGAGGAGAC | CGCCTGGCTGTAGACGAAGTAGA |
